# Multi-Disciplinary Approach to Skull Base Paragangliomas

**DOI:** 10.3390/brainsci13111533

**Published:** 2023-10-31

**Authors:** Steven D. Curry, Armine Kocharyan, Gregory P. Lekovic

**Affiliations:** 1 House Clinic, Los Angeles, CA 90017, USA; 2 Department of Head and Neck Surgery, University of California Los Angeles Medical Center, Los Angeles, CA 90095, USA; 3 Department of Neurosurgery, University of California Los Angeles Medical Center, Los Angeles, CA 90095, USA

**Keywords:** paraganglioma, glomus tumor, skull base surgery, stereotactic radiosurgery, CyberKnife, microsurgery

## Abstract

The treatment of skull base paragangliomas has moved towards the use of cranial nerve preservation strategies, using radiation therapy and subtotal resection in instances when aiming for gross total resection would be expected to cause increased morbidity compared to the natural history of the tumor itself. The goal of this study was to analyze the role of surgery in patients with skull base paragangliomas treated with CyberKnife stereotactic radiosurgery (SRS) for definitive tumor control. A retrospective review identified 22 patients (median age 65.5 years, 50% female) treated with SRS from 2010–2022. Fourteen patients (63.6%) underwent microsurgical resection. Gross total resection was performed in four patients for tympanic paraganglioma (*n* = 2), contralateral paraganglioma (*n* = 1), and intracranial tumor with multiple cranial neuropathies (*n* = 1). Partial/subtotal resection was performed for the treatment of pulsatile tinnitus and conductive hearing loss (*n* = 6), chronic otitis and otorrhea (*n* = 2), intracranial extension (*n* = 1), or episodic vertigo due to perilymphatic fistula (*n* = 1). Eighteen patients had clinical and imaging follow-up for a mean (SD) of 4.5 (3.4) years after SRS, with all patients having clinical and radiological tumor control and no mortalities. Surgery remains an important component in the multidisciplinary treatment of skull base paraganglioma when considering other outcomes besides local tumor control.

## 1. Introduction

Paragangliomas are rare tumors overall but are the most common tumors of the jugular foramen and middle ear; hence, they are of importance to skull base surgeons [1,2]. Paragangliomas are slow-growing, benign-acting, neuroendocrine neoplasms that develop from the embryonic neural crest. In the vicinity of the skull base, these tumors can develop from cells in the adventitia of the jugular bulb (jugular paraganglioma, glomus jugulare), middle ear (tympanic paraganglioma, glomus tympanicum), or along one of the three ganglia of the vagus nerve (vagal paraganglioma) [3,4]. Patients with temporal bone paragangliomas (jugular or tympanic paraganglioma) commonly present with hearing loss and pulsatile tinnitus [5,6,7,8,9]. Less commonly, patients with jugular paragangliomas can present with symptoms of facial or lower cranial neuropathy that can manifest as dysphonia, dysphagia, shoulder weakness, or tongue hemiparesis [10]. Vagal paragangliomas can be found incidentally on imaging, but they may also present initially with lower cranial nerve deficits [11].

The management of head and neck paragangliomas has traditionally involved surgical excision with a goal of gross total resection. Tympanic paragangliomas (Fisch class A and B) can be managed surgically to achieve tumor control while improving symptoms of hearing loss and pulsatile tinnitus [12,13,14]. The surgical management of larger (Fisch class C and D) jugulotympanic paragangliomas (JTP) is challenging due to the complex anatomy, including the great vessels, cranial nerves, and proximity to intracranial structures. Similarly, the excision of vagal paragangliomas has resulted in high rates of vagus nerve injury leading to persistent dysphagia and dysphonia [15,16,17,18].

Advances in neuroimaging, refinements in radiation therapy, and an interest in less invasive treatment paradigms for these usually benign-acting lesions have led to a movement away from radical removal to achieve gross total resection of skull base paragangliomas towards more conservative approaches focused on functional preservation [19,20,21]. This has led to the use of individualized treatment using one or more modalities during the course of treatment including surgery, radiation therapy, or observation with serial imaging (“wait-and-scan approach”) as part of a multidisciplinary team approach. These modalities can be combined or used sequentially based on contingencies and patient-specific factors including tumor size, growth, and location; cranial nerve function (ipsilateral and contralateral); patient age; and patient preferences. Understanding the utility of these options can aid in the treatment planning of complex cases. Subtotal surgical resection has been used in symptomatic patients, either alone or together with radiotherapy, to manage large JTPs while maintaining the functionality of the lower cranial nerves [22,23,24]. Stereotactic radiosurgery (SRS) has been shown to be effective when used to arrest tumor growth but often does not lead to the improvement of symptoms such as pulsatile tinnitus and hearing loss; hence, there is a need to consider surgical management options in order to achieve the dual goals of tumor control and symptom reduction [25].

Numerous prior studies have examined outcomes including tumor control and cranial nerve function among patients with head and neck paragangliomas treated with either surgery or radiation therapy to contrast the relative merits of these treatment modalities [21,26,27,28]. The goal of this study was to examine treatment management strategies in patients who underwent CyberKnife SRS to determine the role of surgery in the contemporary multidisciplinary treatment of complex skull base paragangliomas.

## 2. Materials and Methods

A retrospective chart review was performed for patients at a tertiary care neurotology clinic who were treated for paragangliomas by the senior author (G.P.L.). The inclusion criteria were patients with tympanic, jugular, vagal paraganglioma, or a combination of these who underwent CyberKnife for definitive management of their tumor from 2010–2022. Patient charts were reviewed, and demographic and clinical data were collected, including sex, age, diagnosis, prior treatment, clinical history and presentation, radiation therapy (treatment modality, dose, and dates treated), pre-operative embolization, surgical management (indication for treatment, operation performed, date treated, extent of resection, surgical pathology), pertinent imaging records, and length of follow-up after treatment. Tumor extent was stratified using the Fisch classification of paragangliomas. Data on outcome measures were collected including, post-surgery and -radiotherapy new or worsening cranial neuropathy or other treatment-related complications, length of follow-up with tumor control, and mortality.

All patients were treated with CyberKnife radiosurgical ablation using a prescription dose of 27 Gy in three fractions. Prior to treatment, an Aquaplast mask was fabricated to immobilize the patient, and thin-section magnetic resonance imaging (MRI) with multiplanar, multisequence reconstructions in the axial, coronal, and sagittal planes performed prior to and following the administration of intravenous contrast and computed tomography (CT) images using a high resolution multidetector CT scanner with post-processed reformations was obtained to delineate the target tumor volume and critical anatomic structures for treatment planning using the CyberKnife Multiplan software (Accuray Incorporated, Sunnyvale, CA, USA).

Patients were followed after treatment with contrast-enhanced MRI and clinical examination. Tumor control was defined as unchanged (<2 mm growth in any dimension) or decreased tumor volume determined by the greatest linear measurements in the craniocaudal, axial, and transverse dimensions on MRI studies and no new or worsening cranial neuropathies identified on clinical examination.

Descriptive statistics were calculated to summarize the patient series. Continuous variables were reported as mean and standard deviation if normally distributed, or median and interquartile range (IQR) if skewed as determined by the Shapiro–Wilk test. Associations between continuous variables were assessed using independent samples *t*-tests. Statistical analysis was performed in R version 4.3.1 (R Foundation for Statistical Computing, Vienna, Austria). A threshold of *p* < 0.05 was considered significant for all statistical tests.

## 3. Results

### 3.1. Patient Demographics and Clinical Characteristics

There were 22 patients who met inclusion criteria and were retrospectively analyzed. The mean (SD) age was 61.2 (16.8) years (range 15 to 83 years), and 50% were female (Table 1).

Nineteen patients were treated for jugulotympanic paragangliomas, including Fisch class B tympanic paragangliomas (*n* = 3), and Fisch class C (*n* = 15) and class D (*n* = 1) jugular paragangliomas. Three patients were treated for vagal paragangliomas. No tumors were found to be functional/secreting.

### 3.2. Surgical Management

**Figure 1:**
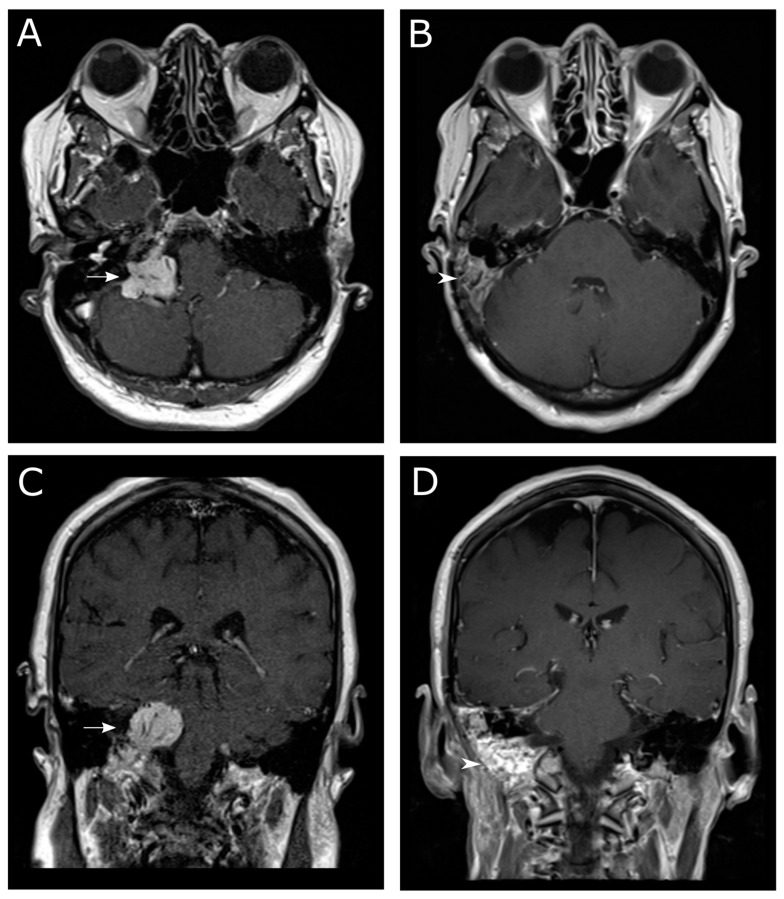
T1-weighted contrast-enhanced MRI of a patient with a Fisch class D jugular paraganglioma who underwent an infratemporal fossa approach for gross total resection of the tumor. The axial pre-surgery image in (**A**) shows brainstem compression from a contrast-enhancing tumor. Part (**B**) shows an axial image slightly more cranial compared to the image in (**A**) showing a fat graft that was used to reconstruct the surgical defect. The coronal pre-surgery image in (**C**) shows contrast-enhancing tumor extending from the jugular bulb to the cerebellopontine angle. The post-surgery image in (**D**) shows the removal of intracranial tumor, with a fat graft visible. Arrows in (**A**,**C**) indicate tumor. Arrowheads in (**B**,**D**) indicate fat graft.

Fourteen patients (63.6%) underwent microsurgical resection. Tympanomastoidectomy with or without an extended facial recess approach was used in half (*n* = 7) of the cases, including all class B tumors (*n* = 3) for both tumor and symptom control, and three class C tumors in cases in which the goal of surgery was relief of otologic symptoms. An infratemporal fossa approach with modifications including with or without closure of the external ear canal, and with or without facial nerve rerouting, was used for the remaining jugular and all cases of vagal paragangliomas. In terms of extent of resection, gross total resection was obtained in four patients for tympanic paraganglioma (*n* = 2), contralateral paraganglioma (*n* = 1), or intracranial tumor with multiple cranial neuropathies (*n* = 1). Figure 1 shows pre- and post-operative imaging results from a patient with a class D tumor with brainstem compression.

**Figure 2:**
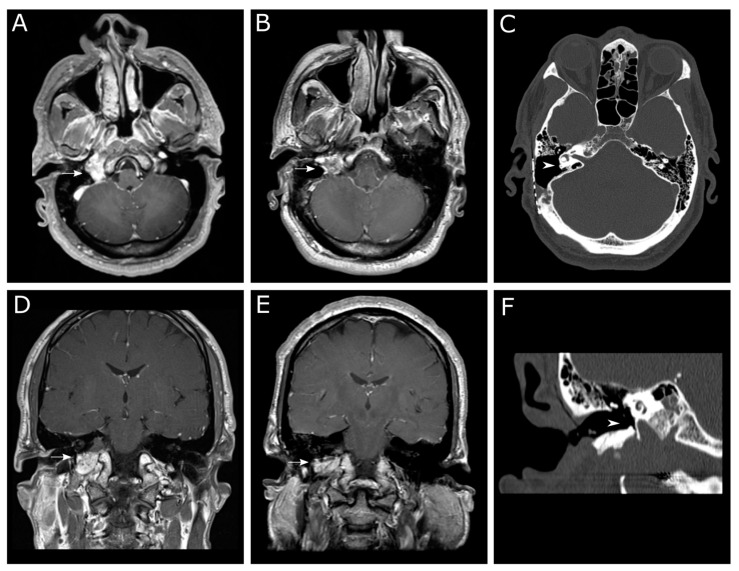
MRI and CT imaging of a patient with a Fisch class C jugular paraganglioma who underwent a modified infratemporal fossa approach for subtotal tumor resection for relief of pulsatile tinnitus and conductive hearing loss. Axial T1-weighted contrast-enhanced MRI presurgical (**A**) and postsurgical (**B**) imaging shows persistent tumor in the area of the jugular bulb. Axial CT bone window (**C**) postsurgical imaging shows no persistent tumor adjacent to the cochlea. Coronal T1-weighted contrast-enhanced MRI presurgical (**D**) and postsurgical (**E**) images show an interval decreased in tumor. Coronal CT bone window (**F**) postsurgical imaging shows no persistent tumor in the mesotympanum or hypotympanum. Arrows in (**A**,**B**,**D**,**E**) indicate tumor. Arrowheads in (**C**,**F**) show the absence of tumor in the middle ear.

Subtotal resection was performed for treatment of pulsatile tinnitus and conductive hearing loss (*n* = 6), chronic otitis and otorrhea (*n* = 2), intracranial extension (*n* = 1), or episodic vertigo due to perilymphatic fistula (*n* = 1). Figure 2 shows imaging results from a patient with a class C tumor who underwent subtotal resection for debilitating pulsatile tinnitus.

Five patients underwent revision surgery for tumor growth or recurrence (*n* = 4) or persistent conductive hearing loss (*n* = 1) following prior infratemporal fossa (*n* = 2) or tympanomastoidectomy (*n* = 3) approaches. This included tumors classified as Fisch class B (*n* = 2), class C (*n* = 2), or class D (*n* = 1) prior to the patients’ first paraganglioma resection. Revision surgical approaches used included tympanomastoidectomy (*n* = 3), infratemporal fossa (*n* = 1), and far lateral (*n* = 1) approaches.

### 3.3. Outcomes

Six patients (43% of patients treated surgically) had new or worsening cranial neuropathies after surgery. Facial nerve palsy occurred in three patients; all improved to House–Brackmann grade 1 or 2. Other cranial neuropathies included worsened dysphonia (*n* = 3) and worsened dysphagia (*n* = 2). No patients required a tracheostomy or gastrostomy.

Patients were treated with SRS a median of 1.4 years (IQR 0.4 to 3.1 years) after initial surgical treatment. The extent of surgical resection (gross total versus subtotal resection) was not associated with a statistically significant difference in time between initial surgery and SRS (*p* = 0.41). Four patients were reported to have new or worsened cranial neuropathies after SRS including dysphagia (*n* = 2), dysphonia (*n* = 1), vertigo (*n* = 1), facial numbness (*n* = 1), and facial spasm (*n* = 1). Eighteen patients had clinical and radiological follow-up for a mean (SD) of 4.5 (3.4) years after SRS, with all patients having clinical and radiological tumor control. There were no mortalities.

## 4. Discussion

Over half (63.6%) of the patients in this series of 22 patients with skull base paragangliomas who were treated with CyberKnife stereotactic radiation for tumor control of skull base paragangliomas underwent surgery. Patients treated with subtotal surgical resection comprised 10 (45.5%) of the series and the majority (10 out of 14, 71.4%) of patients who were treated surgically. Overall, rates of new or worsening cranial neuropathies treatment using approaches directed towards preserving function were much lower compared to the published literature on patients who were treated using surgical approaches focused on gross total resection, thus showing that a management approach ordered towards functional preservation in advanced skull base paragangliomas is practical [15,27,29,30,31].

Despite the typically indolent growth of these tumors, mass effect and local invasion can produce symptoms and warrant management. Operative management of skull base paragangliomas serves a role for alleviating otologic symptoms of conductive hearing loss and pulsatile tinnitus, as well as controlling the effects of secreting tumors, though functional tumors are uncommon among paragangliomas of the head and neck. Surgery may also be indicated in patients with intracranial extension causing brainstem compression or obstructive hydrocephalus [24,32,33,34,35]. The World Health Organization Classification of Head and Neck Tumors now classifies paraganglioma as a tumor of indeterminate biology, rather than benign or malignant, with a spectrum of malignant potential [36]. Known or suspected cases of metastasis may result in additional surgical indications. In the present series, otologic symptoms including pulsatile tinnitus, conductive hearing loss, and external ear canal extension comprised the symptomatic indications for surgery. Other surgical indications in this series included intracranial extension and tympanic paraganglioma treated with an intent to cure. There were no instances of secreting tumors or suspected metastases.

The treatment of skull base paragangliomas is challenging due to the complex anatomy, infiltrative growth though air cell tracts and along foramina and vascular pathways, and the risk of damage to cranial nerves and blood vessels with treatment [25]. The surgical management of jugular foramen tumors can result in high rates of new or worsening cranial nerve deficits, especially to cranial nerves IX to XII as they pass through the jugular foramen and the hypoglossal canal, though lower rates of cranial nerve dysfunction have been reported for surgical management of paragangliomas compared to schwannomas or meningiomas of the jugular foramen [31,37]. The majority of skull base paragangliomas are slow-growing, non-secreting, benign-appearing tumors; thus, it is important that interventions minimize added morbidity and deliver better long-term outcomes than patients would have with the natural course of the disease. These considerations have led to an interest in alternatives to methods such as the infratemporal fossa type A approach, with facial nerve transposition and jugular vein resection, as described by Fisch in 1978 for jugulotympanic paragangliomas [38,39].

Treatment that is more conservative compared to complete surgical extirpation and that encompasses multiple goals, including tumor control, symptom reduction, the relief of brainstem compression if present, and the prevention of late complications while maintaining cranial nerve function, can be individualized based on tumor and patient factors. For patients with symptomatic or growing tumors, subtotal resection with adjuvant or salvage radiation therapy can result in high rates of tumor control with low rates of new or worsening lower cranial neuropathies [27,29,30]. Microsurgical techniques including preservation of the medial wall of the jugular bulb in surgical resection of jugular paragangliomas allow for protection of the lower cranial nerves that pass through the jugular foramen [40,41,42].

Special consideration should be given to paragangliomas of the vagus nerve. Operative management of vagal paragangliomas carries an especially high risk of vagus nerve injury compared to surgery for jugular paragangliomas. In a series of vagal paragangliomas, 37 out of 40 patients treated surgically had sacrifice of the ipsilateral vagus nerve, and all 40 patients had permanent ipsilateral vocal fold paralysis [15]. A systematic review of 226 vagal paragangliomas treated surgically found that the vagus nerve was functionally preserved in only 11 (4.9%) patients [26]. In addition to the laryngeal deficits from vagal nerve injury, high vagal paralysis causes ipsilateral soft palate paralysis and subsequent nasal regurgitation and voice changes. Among elderly or debilitated patients especially, multiple cranial neuropathies, as can occur with aggressive surgical resection of these tumors, may prevent rehabilitation to adequate oral diet [43]. Bilateral vagal nerve palsy can lead to the need for permanent tracheostomy and enteral nutrition. In contrast to these reasons for avoiding surgery, vagal paragangliomas have higher rates of metastasis compared to jugulotympanic paragangliomas [44,45,46]. The management of these tumors should be individualized in light of these considerations.

Radiation therapy, especially SRS with CyberKnife, has emerged as an option for both primary or salvage treatment of skull base paragangliomas [26,28,47]. SRS has excellent rates of tumor control, with decreased morbidity compared to surgery in these cases [21,26]. After a planned subtotal resection for indications including pulsatile tinnitus or conductive hearing loss, SRS can be useful to arrest tumor growth in enlarging tumors, especially in younger patients to reduce the risk of future complications such as cranial neuropathies. In the absence of surgical indications, SRS can be used as a primary therapy to arrest tumor growth. Overall, the incidence of new cranial neuropathy after SRS is low [24,48]. Among patients in our series, two patients had more severe vagal neuropathies (worse dysphagia and/or dysphonia) after SRS, and one patient developed facial numbness and facial spasms after SRS. One important limitation of SRS compared to surgery is that radiation therapy may yield improvement in symptoms in less than half of patients [25].

In the present series, while all patients underwent SRS for definitive control of their paragangliomas (according to the inclusion criteria of this study), most of the patients additionally underwent surgical management for indications including improvement in conductive hearing loss, reducing pulsatile tinnitus, treating otorrhagia and chronic otitis from tumor extension into the external ear canal, and reducing the radiation dose to the cochlea sustained during SRS. Dual-modality treatment (subtotal microsurgical resection with stereotactic radiotherapy) should be considered in patients with bothersome symptoms that are amenable to surgical therapy and who are appropriately counseled regarding the risks and benefits of these treatment modalities.

Angiography with embolization was a commonly used adjunct to surgery. A retrospective study of patients who underwent pre-operative embolization prior to resection of jugular paraganglioma showed a >50% reduction in tumor blush in 86% of patients, with no new or worsening cranial nerve deficits after embolization [49]. Nevertheless, embolization carries risks as the overlapping blood supply between tumors and cranial nerves has been shown to cause facial or lower cranial neuropathies after preoperative embolization with onyx or ethylene vinyl alcohol [50,51]. Cerebrovascular accident can occur due to anastomotic connections between branches of the external carotid artery (e.g., branches of the ascending pharyngeal, deep cervical, ascending cervical, and occipital arteries) and the vertebral artery [52].

Non-operative management should be considered especially in patients who are elderly or who have contralateral lower cranial nerve deficits, poor health or life expectancy, or are unable to tolerate surgery. Observation can be considered in asymptomatic and non-growing tumors to avoid treatment-related morbidities. Reports show that a significant portion of jugular paragangliomas may remain stable in size with observation with serial MRI for years, allowing patients to potentially avoid either surgery or radiation therapy [53,54,55,56].

Other considerations in the comprehensive management of skull base paragangliomas include genetic and biochemical testing. The prevalence of germline mutations in head and neck paragangliomas is now recognized to be about 40%, and the results of genetic testing can provide information for risk stratification. This can include stratifying the risk of aggressive tumor behavior and the development of future tumors, whether metastatic, synchronous, or metachronous paragangliomas, or other tumors that can present as part of a syndrome including renal cell carcinoma, papillary thyroid carcinoma, neuroblastoma, or gastrointestinal stroma tumors [57]. Mutations in subunits of succinate dehydrogenase (SDH) are the most common, and tumors with SDHB mutations have the highest risk for metastasis [36,58,59,60]. Clinical practice and clinical consensus guidelines recommend referral for genetic testing for all patients diagnosed with paragangliomas, and initial biochemical testing with either plasma free metanephrines or urinary fractionated metanephrines is recommended to evaluate for the presence of secreting tumors [57,61,62].

Given the rarity of these tumors, the present study comprises a relatively large number of patients treated with definitive SRS. This series illustrates multiple indications for surgery apart from tumor control, showing that in the multidisciplinary treatment of patients with skull base paragangliomas both surgery and radiation therapy may have value, and the comprehensive treatment of these patients should address both tumor control and symptom amelioration. One of the limitations of this study is that it encompasses a heterogenous group of patients, with a variety of presenting symptoms, tumor locations and sizes, patient ages, and comorbidities. Treatment decisions were determined by shared decision making between surgeons and patients for diverse reasons rather than per a defined protocol. Given these limitations, the ability to quantitatively analyze the outcomes and generalize them to other patient populations may be limited.

## 5. Conclusions

The management of head and neck paragangliomas has evolved to focus on long term preservation of function, with many tumors being treated more conservatively than they would have been in the past, and radiotherapy is a common treatment modality for achieving tumor control. Among patients with skull base paragangliomas who are treated with CyberKnife SRS for definitive management of their tumors, surgery remains an important component in the multidisciplinary treatment when considering other outcomes beyond local tumor control including the treatment of pulsatile tinnitus, conductive hearing loss, chronic otitis and otorrhea, intracranial extension of tumor, or episodic vertigo due to perilymphatic fistula.

## Figures and Tables

**Table 1:** Patient demographic and clinical characteristics.

Variable	Value
Age, mean (SD)	61.2 (16.8) years
Female (%)	11 (50%)
Tumor classification	
Jugulotympanic paraganglioma	19
Fisch class A	0
Fisch class B	3
Fisch class C	15
Fisch class D	1
Vagal paraganglioma	3

Abbreviations: SD, standard deviation.

## Data Availability

Data used for the current original research are available from the corresponding author upon reasonable request.

## References

[1] Wasserman P.G., Savargaonkar P. (2001). Paragangliomas: Classification, Pathology, and Differential Diagnosis. Otolaryngol. Clin. N. Am..

[2] Ramina R., Maniglia J.J., Fernandes Y.B., Paschoal J.R., Pfeilsticker L.N., Coelho Neto M. (2005). Tumors of the Jugular Foramen: Diagnosis and Management. Neurosurgery.

[3] Szymańska A., Szymański M., Czekajska-Chehab E., Gołąbek W., Szczerbo-Trojanowska M. (2015). Diagnosis and Management of Multiple Paragangliomas of the Head and Neck. Eur. Arch. Otorhinolaryngol..

[4] Graham N.J., Smith J.D., Else T., Basura G.J. (2022). Paragangliomas of the Head and Neck: A Contemporary Review. Endocr. Oncol..

[5] Ramos Macías A., Bueno Yanes J., Bolaños Hernández P., Lisner Contreras I., Osorio Acosta A., Vicente Barrero M., Zaballos González M.L. (2011). Temporal paragangliomas. A 12-year experience. Acta Otorrinolaringol. Esp..

[6] Waldvogel D., Mattle H.P., Sturzenegger M., Schroth G. (1998). Pulsatile Tinnitus—A Review of 84 Patients. J. Neurol..

[7] Hofmann E., Behr R., Neumann-Haefelin T., Schwager K. (2013). Pulsatile Tinnitus: Imaging and Differential Diagnosis. Dtsch. Arztebl. Int..

[8] Song J.-J., An G.S., Choi I., De Ridder D., Kim S.Y., Choi H.S., Park J.H., Choi B.Y., Koo J.-W., Lee K. (2016). Objectification and Differential Diagnosis of Vascular Pulsatile Tinnitus by Transcanal Sound Recording and Spectrotemporal Analysis: A Preliminary Study. Otol. Neurotol..

[9] Thomsen K., Elbrond O., Andersen A.P. (1975). Glomus Jugulare Tumours. (A Series of 21 Cases). J. Laryngol. Otol..

[10] Kaylie D.M., O’Malley M., Aulino J.M., Jackson C.G. (2007). Neurotologic Surgery for Glomus Tumors. Otolaryngol. Clin. N. Am..

[11] Offergeld C., Brase C., Yaremchuk S., Mader I., Rischke H.C., Gläsker S., Schmid K.W., Wiech T., Preuss S.F., Suárez C. (2012). Head and Neck Paragangliomas: Clinical and Molecular Genetic Classification. Clinics.

[12] Sanna M., Fois P., Pasanisi E., Russo A., Bacciu A. (2010). Middle Ear and Mastoid Glomus Tumors (Glomus Tympanicum): An Algorithm for the Surgical Management. Auris Nasus Larynx.

[13] Carlson M.L., Sweeney A.D., Pelosi S., Wanna G.B., Glasscock M.E., Haynes D.S. (2015). Glomus Tympanicum: A Review of 115 Cases over 4 Decades. Otolaryngol. Head Neck Surg..

[14] Prasad S.C., Mimoune H.A., Khardaly M., Piazza P., Russo A., Sanna M. (2016). Strategies and Long-Term Outcomes in the Surgical Management of Tympanojugular Paragangliomas. Head Neck.

[15] Netterville J.L., Jackson C.G., Miller F.R., Wanamaker J.R., Glasscock M.E. (1998). Vagal Paraganglioma: A Review of 46 Patients Treated during a 20-Year Period. Arch. Otolaryngol. Head Neck Surg..

[16] Zanoletti E., Mazzoni A. (2006). Vagal Paraganglioma. Skull Base.

[17] Bradshaw J.W., Jansen J.C. (2005). Management of Vagal Paraganglioma: Is Operative Resection Really the Best Option?. Surgery.

[18] Lozano F.S., Gómez J.L., Mondillo M.C., González-Porras J.R., González-Sarmiento R., Muñoz A. (2008). Surgery of Vagal Paragangliomas: Six Patients and Review of Literature. Surg. Oncol..

[19] Foote R.L., Pollock B.E., Gorman D.A., Schomberg P.J., Stafford S.L., Link M.J., Kline R.W., Strome S.E., Kasperbauer J.L., Olsen K.D. (2002). Glomus Jugulare Tumor: Tumor Control and Complications after Stereotactic Radiosurgery. Head Neck.

[20] Mazzoni A., Zanoletti E. (2016). Observation and Partial Targeted Surgery in the Management of Tympano-Jugular Paraganglioma: A Contribution to the Multioptional Treatment. Eur. Arch. Otorhinolaryngol..

[21] Campbell J.C., Lee J.W., Ledbetter L., Wick C.C., Riska K.M., Cunningham C.D., Russomando A.C., Truong T., Hong H., Kuchibhatla M. (2023). Systematic Review and Meta-Analysis for Surgery Versus Stereotactic Radiosurgery for Jugular Paragangliomas. Otol. Neurotol..

[22] Willen S.N., Einstein D.B., Maciunas R.J., Megerian C.A. (2005). Treatment of Glomus Jugulare Tumors in Patients with Advanced Age: Planned Limited Surgical Resection Followed by Staged Gamma Knife Radiosurgery: A Preliminary Report. Otol. Neurotol..

[23] Cosetti M., Linstrom C., Alexiades G., Tessema B., Parisier S. (2008). Glomus Tumors in Patients of Advanced Age: A Conservative Approach. Laryngoscope.

[24] Wanna G.B., Sweeney A.D., Carlson M.L., Latuska R.F., Rivas A., Bennett M.L., Netterville J.L., Haynes D.S. (2014). Subtotal Resection for Management of Large Jugular Paragangliomas with Functional Lower Cranial Nerves. Otolaryngol. Head Neck Surg..

[25] Miller J.P., Semaan M.T., Maciunas R.J., Einstein D.B., Megerian C.A. (2009). Radiosurgery for Glomus Jugulare Tumors. Otolaryngol. Clin. N. Am..

[26] Suárez C., Rodrigo J.P., Bödeker C.C., Llorente J.L., Silver C.E., Jansen J.C., Takes R.P., Strojan P., Pellitteri P.K., Rinaldo A. (2013). Jugular and Vagal Paragangliomas: Systematic Study of Management with Surgery and Radiotherapy. Head Neck.

[27] Ivan M.E., Sughrue M.E., Clark A.J., Kane A.J., Aranda D., Barani I.J., Parsa A.T. (2011). A Meta-Analysis of Tumor Control Rates and Treatment-Related Morbidity for Patients with Glomus Jugulare Tumors. J. Neurosurg..

[28] Lieberson R.E., Adler J.R., Soltys S.G., Choi C., Gibbs I.C., Chang S.D. (2012). Stereotactic Radiosurgery as the Primary Treatment for New and Recurrent Paragangliomas: Is Open Surgical Resection Still the Treatment of Choice?. World Neurosurg..

[29] Guss Z.D., Batra S., Limb C.J., Li G., Sughrue M.E., Redmond K., Rigamonti D., Parsa A.T., Chang S., Kleinberg L. (2011). Radiosurgery of Glomus Jugulare Tumors: A Meta-Analysis. Int. J. Radiat. Oncol. Biol. Phys..

[30] Manzoor N.F., Yancey K.L., Aulino J.M., Sherry A.D., Khattab M.H., Cmelak A., Morrel W.G., Haynes D.S., Bennett M.L., O’Malley M.R. (2021). Contemporary Management of Jugular Paragangliomas with Neural Preservation. Otolaryngol. Head Neck Surg..

[31] Fayad J.N., Keles B., Brackmann D.E. (2010). Jugular Foramen Tumors: Clinical Characteristics and Treatment Outcomes. Otol. Neurotol..

[32] Sanna M., Jain Y., De Donato G., Rohit, Lauda L., Taibah A. (2004). Management of Jugular Paragangliomas: The Gruppo Otologico Experience. Otol. Neurotol..

[33] Fayad J.N., Schwartz M.S., Brackmann D.E. (2009). Treatment of Recurrent and Residual Glomus Jugulare Tumors. Skull Base.

[34] Carlson M.L., Driscoll C.L.W., Garcia J.J., Janus J.R., Link M.J. (2012). Surgical Management of Giant Transdural Glomus Jugulare Tumors with Cerebellar and Brainstem Compression. J. Neurol. Surg. B Skull Base.

[35] Moe K.S., Li D., Linder T.E., Schmid S., Fisch U. (1999). An Update on the Surgical Treatment of Temporal Bone Paraganglioma. Skull Base Surg..

[36] Williams M.D., Tischler A.S. (2017). Update from the 4th Edition of the World Health Organization Classification of Head and Neck Tumours: Paragangliomas. Head Neck Pathol..

[37] Lustig L.R., Jackler R.K. (1996). The Variable Relationship between the Lower Cranial Nerves and Jugular Foramen Tumors: Implications for Neural Preservation. Am. J. Otol..

[38] Fisch U. (1978). Infratemporal Fossa Approach to Tumours of the Temporal Bone and Base of the Skull. J. Laryngol. Otol..

[39] Fisch U., Fagan P., Valavanis A. (1984). The Infratemporal Fossa Approach for the Lateral Skull Base. Otolaryngol. Clin. N. Am..

[40] Al-Mefty O., Fox J.L., Rifai A., Smith R.R. (1987). A Combined Infratemporal and Posterior Fossa Approach for the Removal of Giant Glomus Tumors and Chondrosarcomas. Surg. Neurol..

[41] Al-Mefty O., Teixeira A. (2002). Complex Tumors of the Glomus Jugulare: Criteria, Treatment, and Outcome. J. Neurosurg..

[42] de Brito R., Cisneros Lesser J.C., Lopes P.T., Bento R.F. (2018). Preservation of the Facial and Lower Cranial Nerves in Glomus Jugulare Tumor Surgery: Modifying Our Surgical Technique for Improved Outcomes. Eur. Arch. Otorhinolaryngol..

[43] Netterville J.L., Civantos F.J. (1993). Rehabilitation of Cranial Nerve Deficits after Neurotologic Skull Base Surgery. Laryngoscope.

[44] Heinrich M.C., Harris A.E., Bell W.R. (1985). Metastatic Intravagal Paraganglioma. Case Report and Review of the Literature. Am. J. Med..

[45] Browne J.D., Fisch U., Valavanis A. (1993). Surgical Therapy of Glomus Vagale Tumors. Skull Base Surg..

[46] González-Orús Álvarez-Morujo R., Arístegui Ruiz M., Martin Oviedo C., Álvarez Palacios I., Scola Yurrita B. (2015). Management of Vagal Paragangliomas: Review of 17 Patients. Eur. Arch. Otorhinolaryngol..

[47] Sager O., Dincoglan F., Beyzadeoglu M. (2015). Stereotactic Radiosurgery of Glomus Jugulare Tumors: Current Concepts, Recent Advances and Future Perspectives. CNS Oncol..

[48] Elshaikh M.A., Mahmoud-Ahmed A.S., Kinney S.E., Wood B.G., Lee J.H., Barnett G.H., Suh J.H. (2002). Recurrent Head-and-Neck Chemodectomas: A Comparison of Surgical and Radiotherapeutic Results. Int. J. Radiat. Oncol. Biol. Phys..

[49] Helal A., Vakharia K., Brinjikji W., Carlson M.L., Driscoll C.L., Van Gompel J.J., Link M.J., Cloft H. (2022). Preoperative Embolization of Jugular Paraganglioma Tumors Using Particles Is Safe and Effective. Interv. Neuroradiol..

[50] Gaynor B.G., Elhammady M.S., Jethanamest D., Angeli S.I., Aziz-Sultan M.A. (2014). Incidence of Cranial Nerve Palsy after Preoperative Embolization of Glomus Jugulare Tumors Using Onyx. J. Neurosurg..

[51] Gartrell B.C., Hansen M.R., Gantz B.J., Gluth M.B., Mowry S.E., Aagaard-Kienitz B.L., Baskaya M.K., Gubbels S.P. (2012). Facial and Lower Cranial Neuropathies after Preoperative Embolization of Jugular Foramen Lesions with Ethylene Vinyl Alcohol. Otol. Neurotol..

[52] De Marini P., Greget M., Boatta E., Jahn C., Enescu I., Garnon J., Dalili D., Cazzato R.L., Gangi A. (2021). Safety and Technical Efficacy of Pre-Operative Embolization of Head and Neck Paragangliomas: A 10-Year Mono-Centric Experience and Systematic Review. Clin. Imaging.

[53] van der Mey A.G., Frijns J.H., Cornelisse C.J., Brons E.N., van Dulken H., Terpstra H.L., Schmidt P.H. (1992). Does Intervention Improve the Natural Course of Glomus Tumors? A Series of 108 Patients Seen in a 32-Year Period. Ann. Otol. Rhinol. Laryngol..

[54] Jansen J.C., van den Berg R., Kuiper A., van der Mey A.G., Zwinderman A.H., Cornelisse C.J. (2000). Estimation of Growth Rate in Patients with Head and Neck Paragangliomas Influences the Treatment Proposal. Cancer.

[55] Prasad S.C., Mimoune H.A., D’Orazio F., Medina M., Bacciu A., Mariani-Costantini R., Piazza P., Sanna M. (2014). The Role of Wait-and-Scan and the Efficacy of Radiotherapy in the Treatment of Temporal Bone Paragangliomas. Otol. Neurotol..

[56] Carlson M.L., Sweeney A.D., Wanna G.B., Netterville J.L., Haynes D.S. (2015). Natural History of Glomus Jugulare: A Review of 16 Tumors Managed with Primary Observation. Otolaryngol. Head Neck Surg..

[57] Lloyd S., Obholzer R., Tysome J. (2020). BSBS Consensus Group British Skull Base Society Clinical Consensus Document on Management of Head and Neck Paragangliomas. Otolaryngol. Head Neck Surg..

[58] Favier J., Amar L., Gimenez-Roqueplo A.-P. (2015). Paraganglioma and Phaeochromocytoma: From Genetics to Personalized Medicine. Nat. Rev. Endocrinol..

[59] Cass N.D., Schopper M.A., Lubin J.A., Fishbein L., Gubbels S.P. (2020). The Changing Paradigm of Head and Neck Paragangliomas: What Every Otolaryngologist Needs to Know. Ann. Otol. Rhinol. Laryngol..

[60] Baysal B.E., Willett-Brozick J.E., Lawrence E.C., Drovdlic C.M., Savul S.A., McLeod D.R., Yee H.A., Brackmann D.E., Slattery W.H., Myers E.N. (2002). Prevalence of SDHB, SDHC, and SDHD Germline Mutations in Clinic Patients with Head and Neck Paragangliomas. J. Med. Genet..

[61] Lenders J.W.M., Duh Q.-Y., Eisenhofer G., Gimenez-Roqueplo A.-P., Grebe S.K.G., Murad M.H., Naruse M., Pacak K., Young W.F. (2014). Endocrine Society Pheochromocytoma and Paraganglioma: An Endocrine Society Clinical Practice Guideline. J. Clin. Endocrinol. Metab..

[62] Taïeb D., Wanna G.B., Ahmad M., Lussey-Lepoutre C., Perrier N.D., Nölting S., Amar L., Timmers H.J.L.M., Schwam Z.G., Estrera A.L. (2023). Clinical Consensus Guideline on the Management of Phaeochromocytoma and Paraganglioma in Patients Harbouring Germline SDHD Pathogenic Variants. Lancet Diabetes Endocrinol..

